# Contribution of Dysferlin Deficiency to Skeletal Muscle Pathology in Asymptomatic and Severe Dystroglycanopathy Models: Generation of a New Model for Fukuyama Congenital Muscular Dystrophy

**DOI:** 10.1371/journal.pone.0106721

**Published:** 2014-09-08

**Authors:** Motoi Kanagawa, Zhongpeng Lu, Chiyomi Ito, Chie Matsuda, Katsuya Miyake, Tatsushi Toda

**Affiliations:** 1 Division of Neurology/Molecular Brain Science, Kobe University Graduate School of Medicine, Kobe, Japan; 2 Biomedical Research Institute, National Institute of Advanced Industrial Science and Technology, Tsukuba, Japan; 3 Department of Histology and Cell Biology, School of Medicine, Kagawa University, Ikenobe, Miki, Kagawa, Japan; Rutgers University -New Jersey Medical School, United States of America

## Abstract

Defects in dystroglycan glycosylation are associated with a group of muscular dystrophies, termed dystroglycanopathies, that include Fukuyama congenital muscular dystrophy (FCMD). It is widely believed that abnormal glycosylation of dystroglycan leads to disease-causing membrane fragility. We previously generated knock-in mice carrying a founder retrotransposal insertion in *fukutin*, the gene responsible for FCMD, but these mice did not develop muscular dystrophy, which hindered exploring therapeutic strategies. We hypothesized that dysferlin functions may contribute to muscle cell viability in the knock-in mice; however, pathological interactions between glycosylation abnormalities and dysferlin defects remain unexplored. To investigate contributions of dysferlin deficiency to the pathology of dystroglycanopathy, we have crossed dysferlin-deficient *dysferlin* ^sjl/sjl^ mice to the fukutin-knock-in *fukutin* ^Hp/−^ and Large-deficient *Large* ^myd/myd^ mice, which are phenotypically distinct models of dystroglycanopathy. The *fukutin* ^Hp/−^ mice do not show a dystrophic phenotype; however, (*dysferlin* ^sjl/sjl^: *fukutin* ^Hp/−^) mice showed a deteriorated phenotype compared with (*dysferlin* ^sjl/sjl^: *fukutin* ^Hp/+^) mice. These data indicate that the absence of functional dysferlin in the asymptomatic *fukutin* ^Hp/−^ mice triggers disease manifestation and aggravates the dystrophic phenotype. A series of pathological analyses using double mutant mice for Large and dysferlin indicate that the protective effects of dysferlin appear diminished when the dystrophic pathology is severe and also may depend on the amount of dysferlin proteins. Together, our results show that dysferlin exerts protective effects on the *fukutin* ^Hp/−^ FCMD mouse model, and the (*dysferlin* ^sjl/sjl^: *fukutin* ^Hp/−^) mice will be useful as a novel model for a recently proposed antisense oligonucleotide therapy for FCMD.

## Introduction

Muscular dystrophies are a heterogeneous group of genetic disorders characterized by the progressive loss of muscle strength and integrity. Several lines of evidence have established that the structural linkage between the muscle extracellular matrix and the cytoskeleton is essential in preventing the progression of muscular dystrophy [1]. The dystrophin-glycoprotein complex (DGC) forms the structural linkage, and mutations in components of this complex cause several forms of muscular dystrophy, including Duchenne and limb-girdle muscular dystrophies (LGMDs) [2]. Within the DGC, α- and β-dystroglycans (DG) act as a molecular bridge between the extracellular matrix and the cytoskeleton. α-DG is a highly glycosylated extracellular subunit that functions as a receptor for extracellular matrix proteins such as laminins. *O*-mannosyl glycosylation and a novel phosphodiester-linked modification of *O*-mannose, termed post-phosphoryl modification, are necessary for α-DG to serve as a functional laminin receptor [3], [4]. α-DG is anchored on the plasma membrane through non-covalent interaction with a transmembrane-type β-DG, which in turn binds to the dystrophin-actin cytoskeleton.

Fukuyama congenital muscular dystrophy (FCMD: MIM 253800) is an autosomal recessive disorder characterized by severe muscular dystrophy, abnormal neuronal migration associated with mental retardation and, frequently, eye abnormalities [5]. We identified *fukutin*, the gene responsible for FCMD, and a 3-kb SINE-VNTR-*Alu* (SVA) retrotransposon insertion into the 3′ UTR of *fukutin* as the founder mutation in FCMD [6]. This insertion causes abnormal splicing that leads to the production of non-functional fukutin protein [7]. The introduction of antisense oligonucleotides that target the splice acceptor and splicing enhancers prevented the pathogenic abnormal splicing by SVA in the cells of FCMD patients as well as model mice that carry the retrotransposal insertion [7]. Point mutations in *fukutin* have been reported in patients both inside and outside Japan, and recent studies have revealed a broad clinical spectrum for fukutin-deficient muscular dystrophies [8]. In FCMD, α-DG is abnormally glycosylated, and its laminin-binding activity is decreased [3]. Several other forms of muscular dystrophy are caused by abnormal glycosylation of α-DG; collectively, these conditions are termed “dystroglycanopathies”. More than 10 genes have been identified as causative genes in dystroglycanopathies [9]–[14], some of which encode products that possess enzyme activities involved in synthesizing *O*-mannosyl sugar chains on α-DG [15]–[18]. Fukutin, LARGE, and Fukutin-related protein (FKRP) participate in forming the post-phosphoryl moiety [4], [19]. Overall, dystroglycanopathy gene products appear to be involved in *O*-mannosyl chain synthesis and post-phosphoryl modification; mutations in these pathways commonly result in abnormal glycosylation of α-DG and reduced ligand-binding activity, disrupting the DG-mediated linkage between the extracellular matrix and the cytoskeleton [2].

Defects in DGC components or α-DG glycosylation disrupt the linkage between the extracellular matrix and the cytoskeleton, thus rendering the sarcolemma more susceptible to contraction-induced damage. This is thought to trigger an increase in intracellular Ca^2+^ concentration, eventually leading to necrosis and myofiber degeneration. Myofibers possess an intrinsic mechanism for repair of damaged membranes, and dysferlin plays a pivotal role in the skeletal muscle membrane repair pathway. In humans, dysferlin deficiency leads to LGMD2B, Miyoshi myopathy or a distal myopathy with anterior tibial onset [20]. Dysferlin-deficient mice show defective membrane repair and also develop muscular dystrophy [21]. Several proteins are known to interact with dysferlin [20], and it is expected that these proteins also participate in membrane repair. For example, mitsugumin 53 (MG53, also known as TRIM72) has been implicated in vesicle trafficking to the damage site during the membrane repair process [22].

We previously described a new FCMD mouse model that carries the retrotransposal insertion in the mouse *fukutin* ortholog [23]. These knock-in mice exhibit hypoglycosylated α-DG but do not develop muscular dystrophy. Therefore, these mice are not suitable for testing effectiveness of the antisense oligonucleotide therapy for FCMD. Although skeletal muscle-selective fukutin conditional knock-out mice, namely MCK-fukutin-cKO and Myf5-fukutin-cKO, show dystrophic phenotype [24], they are not applicable for the examination of the antisense oligonucleotide therapy because they do not possess the retrotransposal insertion. We previously reported that the small amount of normally glycosylated α-DG remaining in the skeletal muscle of the knock-in mice prevents muscular dystrophy [23]. However, it is not clear whether this residual glycosylation alone is sufficient to maintain skeletal muscle membrane integrity. We hypothesized that dysferlin functions compensate for presumed membrane fragility caused by a reduced interaction between α-DG and laminin. Furthermore, the exact contribution of dysferlin and dysferlin-interacting proteins to the pathology of dystroglycanopathy is not known. To investigate this question, we crossed dysferlin-deficient mice with two distinct dystroglycanopathy mouse models and analyzed the resultant phenotypes. In addition, if the double mutant mice carrying the retrotransposal insertion show worse dystrophic phenotype than those of dysferlin mutant mice, they can be the first model for the novel antisense oligonucleotide therapy for FCMD.

## Materials and Methods

### Animals

Dysferlin-deficient SJL/J mice, a strain with a large deletion in the *Dysf* gene [25], were purchased from Charles River Japan. The transgenic mouse carrying a neo cassette disruption of one *fukutin* allele (*fukutin* ^+/−^) [26] and the transgenic knock-in homozygous mutant mouse carrying the retrotransposal insertion in the mouse *fukutin* ortholog (*fukutin* ^Hp/Hp^) have been described previously [23]. Genotyping for the *Dysf* mutant allele and the *fukutin* mutant allele was performed as described previously [23], [25]. All animal procedures were approved by the Animal Care and Use Committee of Kobe University Graduate School of Medicine (P120202-R2) in accordance with guidelines of Ministry of Education, Culture, Sports, Science and Technology (MEXT) and Japan Society for the Promotion of Science (JSPS). The animals were housed in cages (2–4 mice per cage) with wood-chip bedding in an environmentally controlled room (25°C, 12 h light-dark cycle) and provided food and water *ad libitum* at the animal facility of Kobe University Graduate School of Medicine. Well-trained and skilled researchers and experimental technicians, who have knowledge of methods to prevent unnecessary excessive pain, handled the animals and carried out the experiments. Euthanization was done by cervical dislocation. At sacrifice, the muscles were harvested and snap-frozen in liquid nitrogen (for biochemistry) or in liquid-nitrogen-cooled isopentane (for immunofluorescence and histology). The number and ages of animals used in each experiment is indicated in Figure legends and graphs.

**Figure 1:**
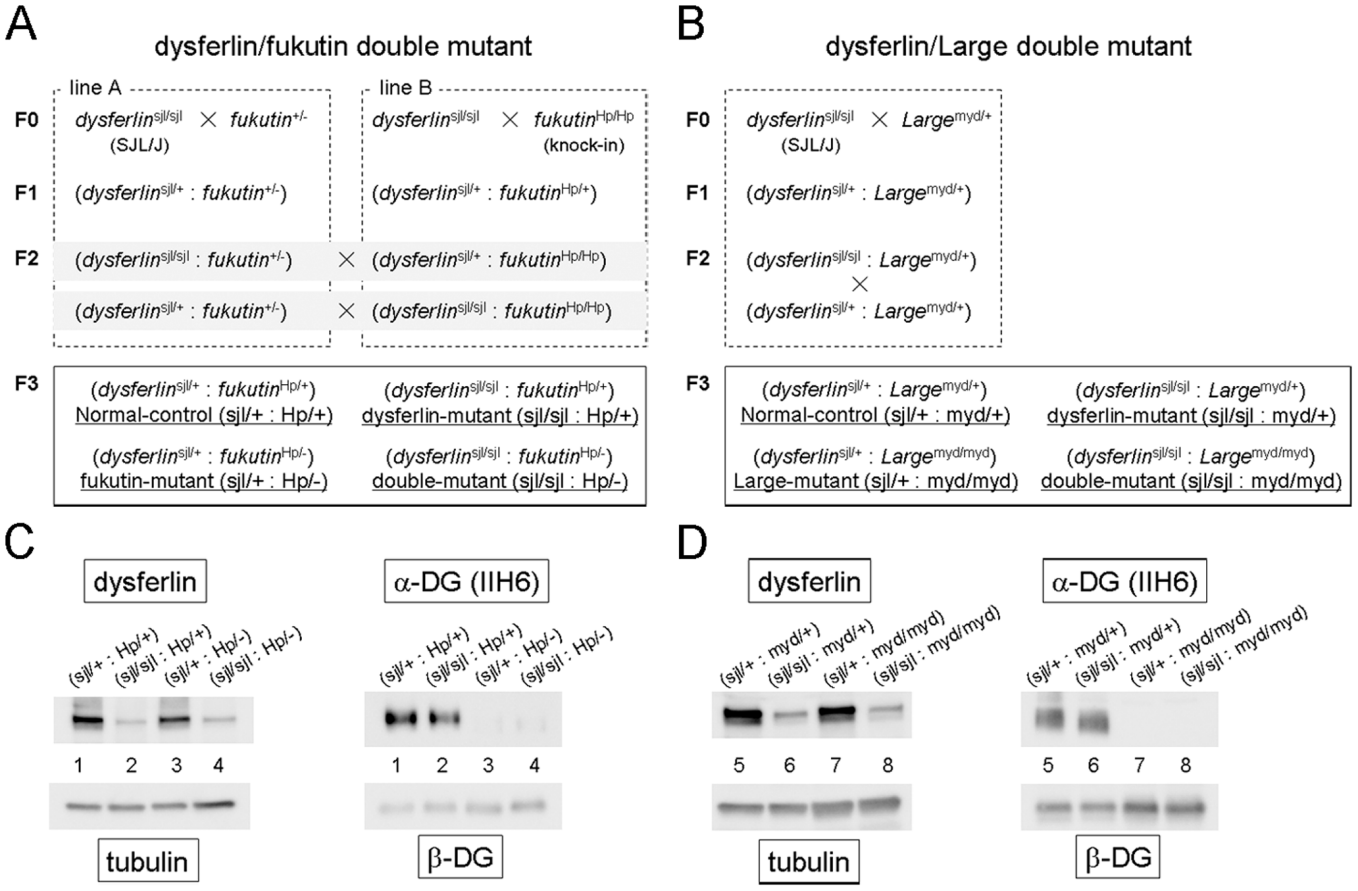
Generation of double-mutant mice exhibiting both abnormal α-DG glycosylation and reduced dysferlin expression. (A, B) Breeding strategy for the generation of double-mutant mice. sjl represents the *dysferlin* mutant allele, myd represents the *Large* mutant allele, and Hp represents the transgenic allele carrying the retrotransposal insertion in *fukutin*. Hp/+ represents a carrier with the insertion in *fukutin*. Hp/− represents a compound heterozygote carrying the insertion and a neo-disrupted allele. For the dysferlin/fukutin double mutant line, we used mice carrying *dysferlin* ^sjl/+^ and *fukutin* ^Hp/+^ as the normal control (*dysferlin* ^sjl/+^: *fukutin* ^Hp/+^); *dysferlin* ^sjl/sjl^ and *fukutin* ^Hp/+^ as the *dysferlin*-mutant (*dysferlin* ^sjl/sjl^: *fukutin* ^Hp/+^); *dysferlin* ^sjl/+^ and *fukutin* ^Hp/−^ as the *fukutin*-mutant (*dysferlin* ^sjl/+^: *fukutin* ^Hp/−^); and *dysferlin* ^sjl/sjl^ and *fukutin* ^Hp/−^ as the double-mutant (*dysferlin* ^sjl/sjl^: *fukutin* ^Hp/−^). For the dysferlin/Large double mutant line, we used mice carrying *dysferlin* ^sjl/+^ and *Large* ^myd/+^ as the normal control (*dysferlin* ^sjl/+^: *Large* ^myd/+^); *dysferlin* ^sjl/sjl^ and *Large* ^myd/+^ as the *dysferlin*-mutant (*dysferlin* ^sjl/sjl^: *Large* ^myd/+^); *dysferlin* ^sjl/+^ and *Large* ^myd/myd^ as the *Large*-mutant (*dysferlin* ^sjl/+^: *Large* ^myd/myd^); and *dysferlin* ^sjl/sjl^ and *Large* ^myd/myd^ as the double mutant (*dysferlin* ^sjl/sjl^: *Large* ^myd/myd^). (C, D) Abnormal α-DG glycosylation and reduced dysferlin protein expression. Solubilized skeletal muscle samples from each genotype were subjected to Western blot analysis for dysferlin protein expression (left panel). Tubulin was used as a loading control. The solubilized fractions were further enriched for DG by WGA-beads, and the DG-enriched fractions were subjected to Western blotting with the monoclonal IIH6 antibody, which recognizes glycosylated α-DG (right panel). β-DG was used as a loading control. The (*dysferlin* ^sjl/+^: *fukutin* ^Hp/+^), (*dysferlin* ^sjl/sjl^: *fukutin* ^Hp/+^), (*dysferlin* ^sjl/+^: *fukutin* ^Hp/−^), and (*dysferlin* ^sjl/sjl^: *fukutin* ^Hp/−^) mice are abbreviated as (sjl/+: Hp/+), (sjl/sjl: Hp/+), (sjl/+: Hp/−), and (sjl/sjl: Hp/−), respectively. The (*dysferlin* ^sjl/+^: *Large* ^myd/+^), (*dysferlin* ^sjl/sjl^: *Large* ^myd/+^), (*dysferlin* ^sjl/+^: *Large* ^myd/myd^), and (*dysferlin* ^sjl/sjl^: *Large* ^myd/myd^) mice are abbreviated as (sjl/+: myd/+), (sjl/sjl: myd/+), (sjl/+: myd/myd), and (sjl/sjl: myd/myd), respectively.

To generate double mutant mice for dysferlin and fukutin deficiency, we crossed dysferlin-deficient SJL/J mice [25] (*dysferlin* ^sjl/sjl^; SJL background) with two different lines of *fukutin* mutant mice. One is a transgenic mouse carrying a neo cassette disruption for a single *fukutin* allele (*fukutin* ^+/−^; 129-C57BL/6 background) [26] (Fig. 1A, line A). The other is a transgenic knock-in homozygous mutant mouse carrying the retrotransposal insertion in the mouse *fukutin* ortholog [23] (*fukutin* ^Hp/Hp^; 129-C57BL/6 background) (Fig. 1A, line B). Heterozygous F1 mice in both lines were intercrossed to obtain the following four genotypes (F2): (*dysferlin* ^sjl/sjl^: *fukutin* ^+/−^); (*dysferlin* ^sjl/+^: *fukutin* ^+/−^); (*dysferlin* ^sjl/+^: *fukutin* ^Hp/Hp^); and (*dysferlin* ^sjl/sjl^: *fukutin* ^Hp/Hp^). We further crossed (*dysferlin* ^sjl/sjl^: *fukutin* ^+/−^) with (*dysferlin* ^sjl/+^: *fukutin* ^Hp/Hp^) mice or (*dysferlin* ^sjl/+^: *fukutin* ^+/−^) with (*dysferlin* ^sjl/sjl^: *fukutin* ^Hp/Hp^) mice (Fig. 1A, highlighted with gray) to produce four genotypes (F3): (*dysferlin* ^sjl/+^: *fukutin* ^Hp/+^); (*dysferlin* ^sjl/+^: *fukutin* ^Hp/−^); (*dysferlin* ^sjl/sjl^: *fukutin* ^Hp/+^); and (*dysferlin* ^sjl/sjl^: *fukutin* ^Hp/−^). To generate double mutant mice for dysferlin and Large deficiency, we crossed dysferlin-deficient SJL/J mice (C57BL/6 backcross 7) with Large-deficient *Large* ^myd^ mice (*Large* ^myd/myd^; C57BL/6 background) [27], [28]. Heterozygous F1 mice were intercrossed and the following four genotypes were used for the analyses (F2): (*dysferlin* ^sjl/+^: *Large* ^myd/+^); (*dysferlin* ^sjl/sjl^: *Large* ^myd/+^); (*dysferlin* ^sjl/+^: *Large* ^myd/myd^); and (*dysferlin* ^sjl/sjl^: *Large* ^myd/myd^). For more effective breeding, we crossed (*dysferlin* ^sjl/+^: *Large* ^myd/+^) mice with (*dysferlin* ^sjl/sjl^: *Large* ^myd/+^) mice (Fig. 1B). (*Dysferlin* ^+/+^: *Large* ^myd/myd^) mice were obtained from the dysferlin/Large double mutant line and *Large* ^myd^ mouse colonies.

### Antibodies

Antibodies used in Western blotting and immunofluorescence were as follows: mouse monoclonal antibody 8D5 against β-DG (Novocastra); mouse monoclonal antibody IIH6 against α-DG (Millipore); affinity-purified goat polyclonal antibody against the α-DG core protein (AP-074G-C) [23]; mouse monoclonal antibody NCL-Hamlet against dysferlin (Novocastra); rat monoclonal antibody against mouse F4/80 (BioLegend); rabbit polyclonal antibody against collagen I (AbD serotec); rabbit polyclonal antibody against albumin (DAKO); mouse monoclonal antibody against caveolin-3 (BD Transduction Laboratories); rabbit polyclonal antibody against caveolin-3 (Abcam); and rabbit polyclonal antibody against Trim72 (MG53) (Abcam).

### Protein preparation and Western blotting

DG was enriched from solubilized skeletal muscle as described previously [23]. Briefly, skeletal muscles were solubilized in Tris-buffered saline (TBS) containing 1% Triton X-100 and protease inhibitors (Nacalai). The solubilized fraction was incubated with wheat germ agglutinin (WGA)-agarose beads (Vector Laboratories) at 4°C for 16 h, and then DG was eluted with SDS-PAGE loading buffer. For detection of dysferlin and dysferlin-interacting proteins, RIPA buffer (1% NP-40, 0.5% DOC, and 0.1% SDS in TBS with protease inhibitors) was used for protein extraction from skeletal muscle. For this experiment, we used *fukutin* ^Hp/−^ mice and litter control *fukutin* ^Hp/+^ mice that were backcrossed to C57BL/6 mice more than 10 times. Protein concentration of the solubilized fractions was measured by Lowry methods, using BSA as a standard. Proteins were separated using 3–15% linear gradient SDS-gels. Gels were transferred to polyvinylidene fluoride (PVDF) membrane (Millipore). Blots were probed with antibodies and then developed with horseradish peroxidase (HRP)-enhanced chemiluminescence (Supersignal West Pico, Pierce; or ECL Plus, GE Healthcare). Protein bands were detected using the LAS-4000 system (Fujifilm), and band intensities were quantified using Multi Gauge V3.2 software (Fujifilm). Statistical analysis was performed with a two-tailed unpaired *t* test. A *p* value of <0.05 was considered to be significant.

### Histological and Immunofluorescence analysis

For H&E staining, cryosections (7 µm) were stained for 2 min in hematoxylin, 1 min in eosin, and then dehydrated with ethanol and xylenes. For Masson trichrome staining, sections were fixed with Bouin's solution (Sigma) for 1 hour at 60°C. The slides were incubated in solution A (5% trichloroacetic acid, 5% potassium dichromate) for 30 min, and then stained with Weigert's iron hematoxylin (Muto Chemical Co Ltd) for 15 min. After a rinse with 0.5% HCl in 70% ethanol and a subsequent rinse with warm water, the slides were incubated in solution B (0.5% phosphotungstic acid, 2.5% phosphomolybdic acid) for 1 min, and then stained with FUCHSIN-PONCEAU solution. The slides were washed with 1% acetic acid, incubated in 2.5% phosphomolybdic acid for 5 min, washed with 1% acetic acid, stained with aniline blue, washed with 1% acetic acid, dehydrated, and mounted.

For immunofluorescence analysis, sections were treated with cold ethanol/acetone (1∶1) for 1 min, blocked with 5% goat serum in MOM Mouse Ig Blocking Reagent (Vector Laboratories) at room temperature for 1 h, and then incubated with primary antibodies diluted in MOM Diluent (Vector Laboratories) overnight at 4°C. The slides were washed with PBS and incubated with Alexa Fluor 488-conjugated or Alexa Fluor 555-conjugated secondary antibodies (Molecular Probes) at room temperature for 30 min. Permount (Fisher Scientific) and TISSU MOUNT (Shiraimatsu Kikai) were used for H&E staining and immunofluorescence, respectively. Sections were observed under fluorescence microscopy (Leica DMR, Leica Microsystems).

For quantitative evaluation of muscle pathology, the percentages of myofiber with centrally located nuclei were counted for at least 1,000 fibers for each genotype (n>4). For evaluation of the F4/80-positive and the collagen I-positive area, the immunofluorescence signal was quantitatively measured using Image J software. Statistical analysis was performed using values represent means with standard deviations, and *p* values <0.05 were considered significant (Student's *t*-test and Mann–Whitney U test).

## Results

### Generation of double mutant mice exhibiting both abnormal glycosylation of α-DG and dysferlin deficiency

To generate double mutant mice, we crossed dysferlin-deficient SJL/L mice (*dysferlin* ^sjl/sjl^) [25] with two distinct dystroglycanopathy models, fukutin-deficient or Large-deficient mice. Previously we reported a transgenic knock-in homozygous mutant mouse carrying the retrotransposal insertion in the mouse *fukutin* ortholog (*fukutin* ^Hp/Hp^) [23]. Compound heterozygous mice carrying the retrotransposal insertion and a neo cassette *fukutin* disruption (*fukutin* ^Hp/−^) showed more abnormal glycosylation of α-DG than did mice homozygous for the insertion (*fukutin* ^Hp/Hp^ mice), although *fukutin* ^Hp/−^ mice did show a detectable amount of residual α-DG glycosylation [23]. For the current study, we generated double mutant mice with the (*dysferlin* ^sjl/sjl^: *fukutin* ^Hp/−^) genotype (Fig. 1A). The other dystroglycanopathy model, Large-deficient *Large* ^myd^ mouse (*Large* ^myd/myd^) [27], [28] show abnormal glycosylation with no detectable amount of properly glycosylated α-DG. The ligand binding activity of α-DG in *Large* ^myd/myd^ mice is greatly reduced compared with that in *fukutin* ^Hp/−^ mice [23]. Breeding strategies, genotypes, and abbreviations for these double mutant mice and their controls are shown in Figure 1A and 1B.

To confirm reduced protein expression of dysferlin and abnormal glycosylation of α-DG in these mice, we prepared solubilized fractions from skeletal muscle extracts and enriched for α-DG using wheat germ agglutinin (WGA)-agarose beads. Western blot analysis showed a dramatic reduction of dysferlin protein in skeletal muscle from (*dysferlin* ^sjl/sjl^: *fukutin* ^Hp/+^), (*dysferlin* ^sjl/sjl^: *fukutin* ^Hp/−^), (*dysferlin* ^sjl/sjl^: *Large* ^myd/+^), and (*dysferlin* ^sjl/sjl^: *Large* ^myd/myd^) mice (Fig. 1C and D). We also confirmed a significant reduction of reactivity against the monoclonal antibody IIH6, which recognizes glycosylated epitopes on α-DG that are necessary for laminin binding activity, in (*dysferlin* ^sjl/+^: *fukutin* ^Hp/−^), (*dysferlin* ^sjl/sjl^: *fukutin* ^Hp/−^), (*dysferlin* ^sjl/+^: *Large* ^myd/myd^), and (*dysferlin* ^sjl/sjl^: *Large* ^myd/myd^) (Fig. 1C and D). Overall, these data confirmed the production of model mice with four biochemically distinct genotypes in each double mutant line.

### More severe muscular dystrophy in (*dysferlin^sjl/sjl^*: *fukutin^Hp/−^*) than in (*dysferlin^sjl/sjl^*: *fukutin^Hp/+^*) mice

**Figure 2:**
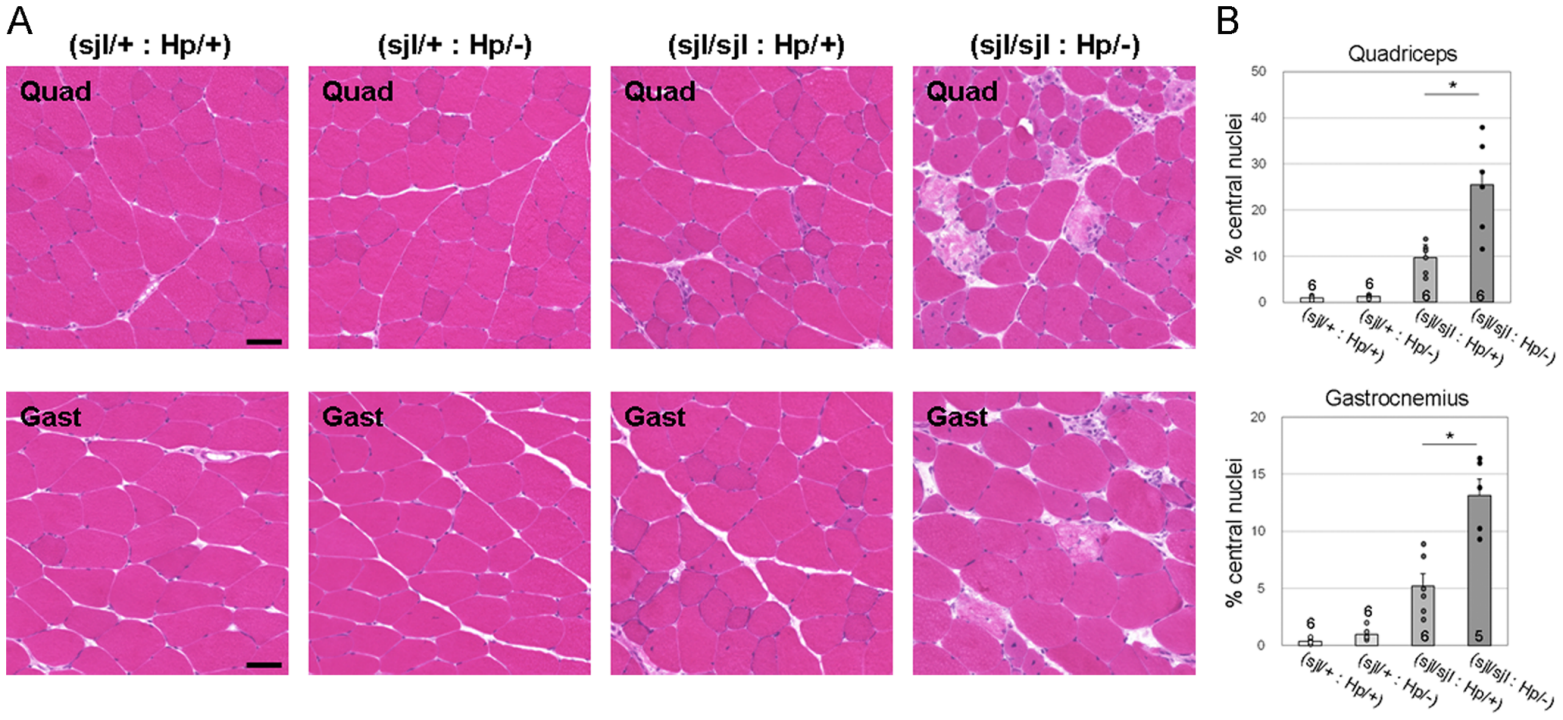
Histological analysis of skeletal muscle from dysferlin/fukutin double mutant mice. (A) Quadriceps (Quad) and gastrocnemius (Gast) muscle tissues from the four mouse genotypes at 15 weeks were analyzed by H&E staining. Bar, 50 µm. (B) Myofibers with centrally located nuclei were counted and quantitatively compared between (*dysferlin* ^sjl/sjl^: *fukutin* ^Hp/+^) and (*dysferlin* ^sjl/sjl^: *fukutin* ^Hp/−^) mice (*, *p*<0.05). Data shown are mean ± s.e.m. for each group (*n* is indicated in the graph). The (*dysferlin* ^sjl/+^: *fukutin* ^Hp/+^), (*dysferlin* ^sjl/+^: *fukutin* ^Hp/−^), (*dysferlin* ^sjl/sjl^: *fukutin* ^Hp/+^), and (*dysferlin* ^sjl/sjl^: *fukutin* ^Hp/−^) mice are abbreviated as (sjl/+: Hp/+), (sjl/+: Hp/−), (sjl/sjl: Hp/+), and (sjl/sjl: Hp/−), respectively.

**Figure 3:**
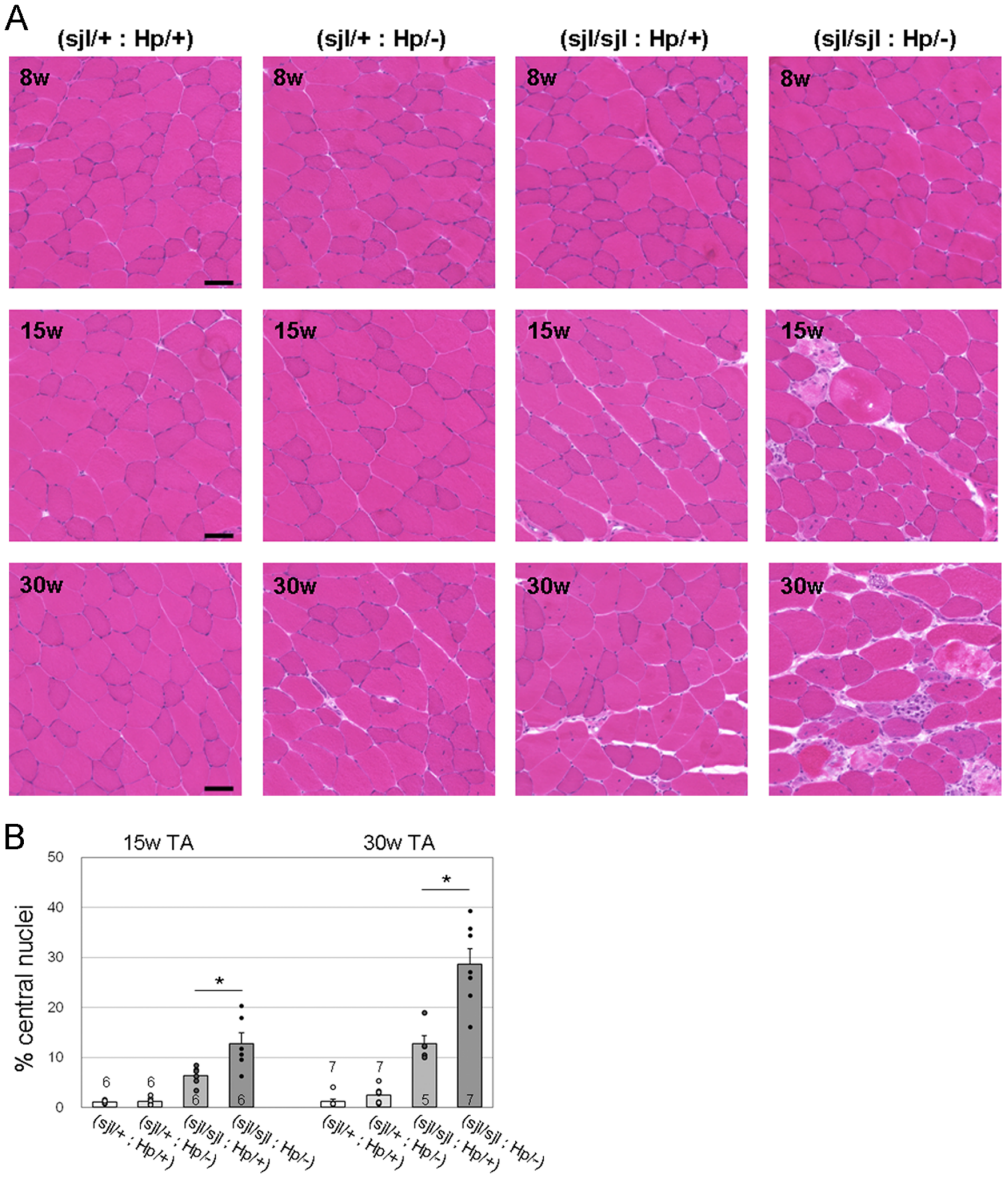
Pathological comparisons between (*dysferlin* ^sjl/sjl^: *fukutin* ^Hp/+^) and (*dysferlin* ^sjl/sjl^ and *fukutin* ^Hp/−^) mice. (A) H&E staining of TA muscle from (*dysferlin* ^sjl/+^: *fukutin* ^Hp/+^), (*dysferlin* ^sjl/+^: *fukutin* ^Hp/−^), (*dysferlin* ^sjl/sjl^: *fukutin* ^Hp/+^) and (*dysferlin* ^sjl/sjl^: *fukutin* ^Hp/−^) mice at 8, 15 and 30 weeks. Bar, 50 µm. (B) Myofibers with centrally located nuclei were counted and quantitatively compared between (*dysferlin* ^sjl/sjl^: *fukutin* ^Hp/+^) and (*dysferlin* ^sjl/sjl^: *fukutin* ^Hp/−^) mice at 15 and 30 weeks (*, *p* < 0.05). Data shown are mean ± s.e.m. for each group (*n* is indicated in the graph). The (*dysferlin* ^sjl/+^: *fukutin* ^Hp/+^), (*dysferlin* ^sjl/+^: *fukutin* ^Hp/−^), (*dysferlin* ^sjl/sjl^: *fukutin* ^Hp/+^), and (*dysferlin* ^sjl/sjl^: *fukutin* ^Hp/−^) mice are abbreviated as (sjl/+: Hp/+), (sjl/+: Hp/−), (sjl/sjl: Hp/+), and (sjl/sjl: Hp/−), respectively.

We examined the histopathology of (*dysferlin* ^sjl/sjl^: *fukutin* ^Hp/−^) mice by hematoxylin and eosin (H&E) staining. The (*dysferlin* ^sjl/+^: *fukutin* ^Hp/−^) mice showed no obvious pathological features of muscular dystrophy (Fig. 2). The (*dysferlin* ^sjl/sjl^: *fukutin* ^Hp/+^) mice showed mild dystrophic changes such as the presence of necrotic fibers and centrally located nuclei (Fig. 2). The phenotypes of (*dysferlin* ^sjl/+^: *fukutin* ^Hp/−^) and (*dysferlin* ^sjl/sjl^: *fukutin* ^Hp/+^) mice are similar to those described previously for retrotransposon knock-in *fukutin* mutant mice and dysferlin-deficient SJL/J mice, respectively [23], [25]. These results also indicate that disruption of one *dysferlin* or one *fukutin* allele does not affect the phenotype of *fukutin* ^Hp/−^ or *dysferlin* ^sjl/sjl^ single mutant mice, respectively. H&E staining showed that the (*dysferlin* ^sjl/sjl^: *fukutin* ^Hp/−^) mice showed further progressed and more severe dystrophic features than did the (*dysferlin* ^sjl/sjl^: *fukutin* ^Hp/+^) mice in quadriceps (Quad), gastrocnemius (Gast), and tibialis anterior (TA) muscles (Fig. 2A and Fig. 3A). Comparison of the percentage of muscle fibers with centrally located nuclei confirmed a more severe dystrophic phenotype in the (*dysferlin* ^sjl/sjl^: *fukutin* ^Hp/−^) mice than that in the (*dysferlin* ^sjl/sjl^: *fukutin* ^Hp/+^) mice (Fig. 2B).

**Figure 4:**
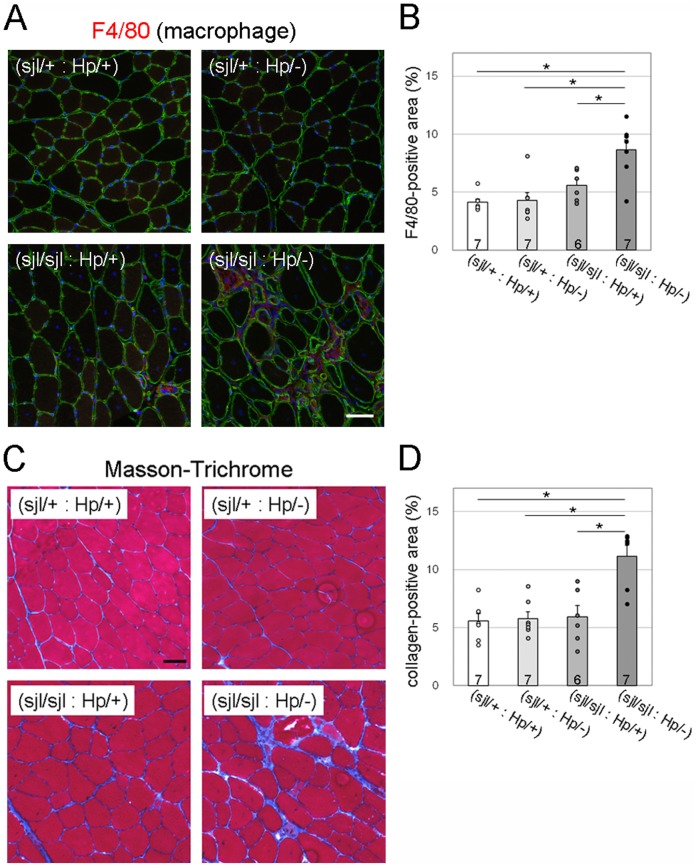
Macrophage and connective tissue infiltration in dysferlin/fukutin double mutant mice. (A) Macrophage infiltration was determined by immunofluorescence analysis using the F4/80 antibody (red). The sarcolemma and nuclei were stained by laminin (green) and DAPI (blue), respectively. TA muscle sections from 30-week-old mice were used. Bar, 50 µm. (B) F4/80-positive immunofluorescence signals were quantified using Image J software. (C) Connective tissue infiltration was determined by Masson-Trichrome staining. TA muscle sections from 30-week-old mice were used. Bar, 50 µm. (D) Quantitative analysis of connective tissue infiltration, determined by immunofluorescence analysis using anti-collagen I antibody. The collagen I-positive area was quantified using Image J software. For quantitative analysis (B and D), data shown are mean ± s.e.m. for each group (*n* is indicated in the graph; *, *p*<0.05). The (*dysferlin* ^sjl/+^: *fukutin* ^Hp/+^), (*dysferlin* ^sjl/+^: *fukutin* ^Hp/−^), (*dysferlin* ^sjl/sjl^: *fukutin* ^Hp/+^), and (*dysferlin* ^sjl/sjl^: *fukutin* ^Hp/−^) mice are abbreviated as (sjl/+: Hp/+), (sjl/+: Hp/−), (sjl/jl: Hp/+), and (sjl/sjl: Hp/−), respectively.

To compare the pathological severity in (*dysferlin* ^sjl/sjl^: *fukutin* ^Hp/−^) and (*dysferlin* ^sjl/sjl^: *fukutin* ^Hp/+^) skeletal muscle more precisely, we counted the percentage of muscle fibers (TA) with centrally located nuclei at different ages (Fig. 3A and B). In 8-week-old mice, we observed a few fibers with centrally located nuclei and necrotic fibers in both the (*dysferlin* ^sjl/sjl^: *fukutin* ^Hp/+^) and the (*dysferlin* ^sjl/sjl^: *fukutin* ^Hp/−^) mice, but no significant differences were seen between the two (data not shown). At 15 weeks and 30 weeks of age, the (*dysferlin* ^sjl/sjl^: *fukutin* ^Hp/−^) mice show significantly more fibers with centrally located nuclei than do the (*dysferlin* ^sjl/sjl^: *fukutin* ^Hp/+^) mice (Fig. 3B). The proportion of fibers with centrally located nuclei in the (*dysferlin* ^sjl/sjl^: *fukutin* ^Hp/−^) mice increased with age. These results indicate more frequent cycles of muscle cell degeneration and regeneration in the (*dysferlin* ^sjl/sjl^: *fukutin* ^Hp/−^) mice. We next compared infiltration of macrophage and connective tissue as indicators of disease severity. Immunofluorescence analysis using the monoclonal F4/80 antibody, a marker for macrophages, indicated that macrophage infiltration was increased in (*dysferlin* ^sjl/sjl^: *fukutin* ^Hp/−^) skeletal muscle compared with (*dysferlin* ^sjl/sjl^: *fukutin* ^Hp/+^) skeletal muscle (Fig. 4A). Quantification of F4/80-immunofluorescence signals confirmed significant increases of macrophage infiltration in (*dysferlin* ^sjl/sjl^: *fukutin* ^Hp/−^) skeletal muscle (Fig. 4B). Masson trichrome staining revealed that the fibrotic area was increased in (*dysferlin* ^sjl/sjl^: *fukutin* ^Hp/−^) skeletal muscle (Fig. 4C). Quantification of immunofluorescence signals for collagen I further supported significant increases of connective tissue infiltrations in (*dysferlin* ^sjl/sjl^: *fukutin* ^Hp/−^) skeletal muscles (Fig. 4D). These data are indicative of further progressed and more severe dystrophic phenotypes in (*dysferlin* ^sjl/sjl^: *fukutin* ^Hp/−^) skeletal muscle. Importantly, although the (*dysferlin* ^sjl/+^: *fukutin* ^Hp/−^) mice do not show muscle pathology, the (*dysferlin* ^sjl/sjl^: *fukutin* ^Hp/−^) mice show a more severe phenotype than do the (*dysferlin* ^sjl/sjl^: *fukutin* ^Hp/+^) mice, suggesting that dysferlin plays a protective role in preventing disease manifestation in the (*dysferlin* ^sjl/+^: *fukutin* ^Hp/−^) mice.

**Figure 5:**
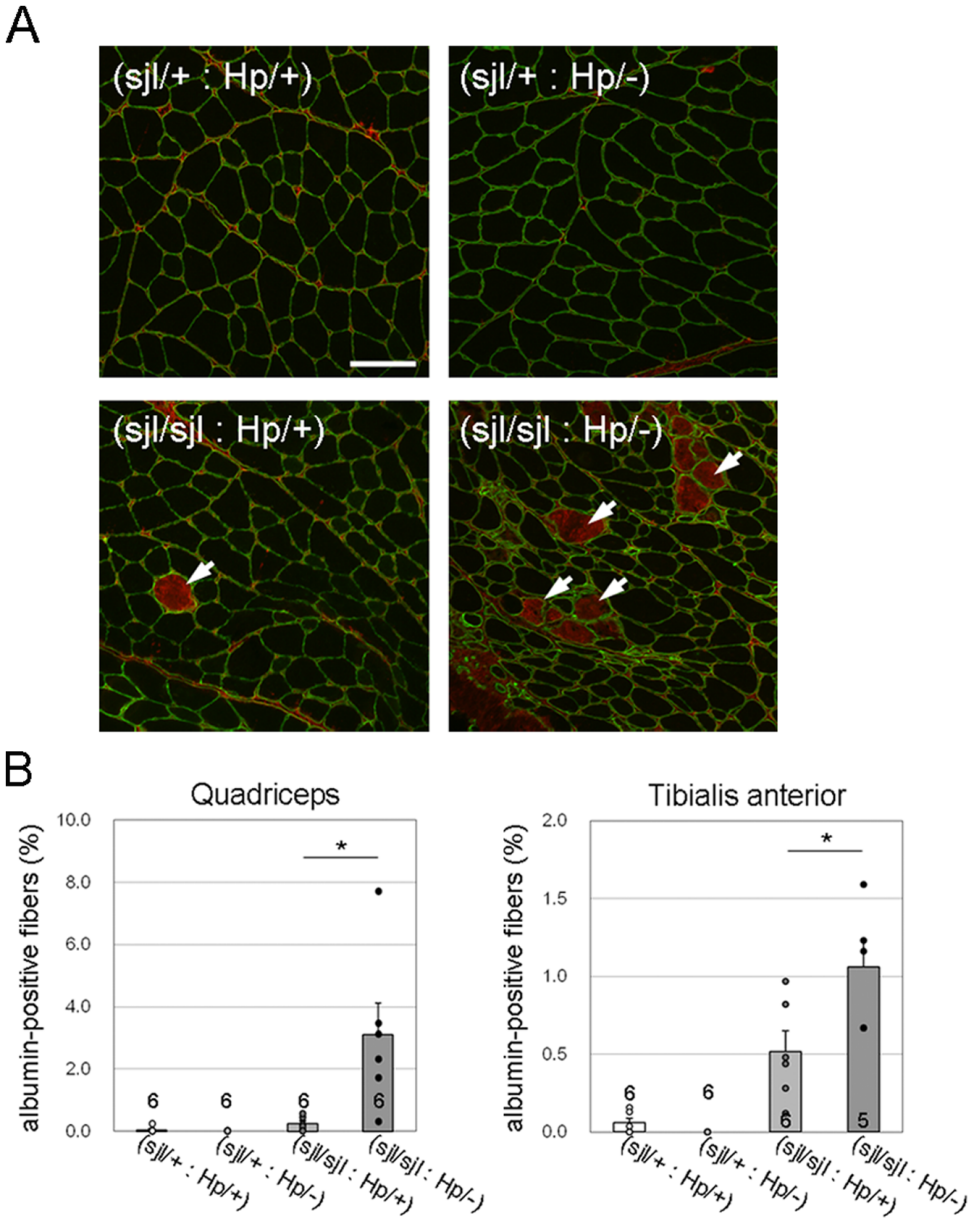
Myofiber membrane fragility in dysferlin/fukutin double mutant mice. (A) Intracellular albumin was determined by immunofluorescence (red). Myofibers are marked by laminin staining (green). Arrows indicate myofibers with intracellular albumin. Images were taken from quadriceps muscle sections of 15-week-old mice. Bar, 100 µm. (B) Myofibers with intracellular albumin were counted and statistically compared between (*dysferlin* ^sjl/sjl^: *fukutin* ^Hp/+^) and (*dysferlin* ^sjl/sjl^: *fukutin* ^Hp/−^) mice. Quadriceps and TA muscle sections from 15-week-old mice were analyzed. Data shown are mean ± s.e.m. for each group (*n* is indicated in the graph; *, *p*<0.05). The (*dysferlin* ^sjl/+^: *fukutin* ^Hp/+^), (*dysferlin* ^sjl/+^: *fukutin* ^Hp/−^), (*dysferlin* ^sjl/sjl^: *fukutin* ^Hp/+^), and (*dysferlin* ^sjl/sjl^: *fukutin* ^Hp/−^) mice are abbreviated as (sjl/+: Hp/+), (sjl/+: Hp/−), (sjl/sjl: Hp/+), and (sjl/sjl: Hp/−), respectively.

Our previous data and those of others suggest that muscle cell membrane fragility due to loss of DG or its functional glycosylation triggers disease manifestation [24], [29]. However, we have not observed evidence indicating membrane fragility in *fukutin* ^Hp/−^ skeletal muscle [23]. To investigate whether membrane fragility is associated mechanistically with the deteriorated phenotype of the (*dysferlin* ^sjl/sjl^: *fukutin* ^Hp/−^) mice, we analyzed the population of albumin-positive muscle fibers. Intracellular albumin staining often is used as an indicator of muscle fiber damage or increased membrane permeability [30]. Immunofluorescence analysis suggested that the albumin-positive myofibers were almost absent in both (*dysferlin* ^sjl/+^: *fukutin* ^Hp/+^) and (*dysferlin* ^sjl/+^: *fukutin* ^Hp/−^) and only sparsely observed in (*dysferlin* ^sjl/sjl^: *fukutin* ^Hp/+^) skeletal muscles, whereas they appeared increased in (*dysferlin* ^sjl/sjl^: *fukutin* ^Hp/−^) skeletal muscle (Fig. 5A). Quantification of albumin-positive fibers also confirmed significant deterioration of the myofiber membrane fragility in the (*dysferlin* ^sjl/sjl^: *fukutin* ^Hp/−^) mice (Fig. 5B). These data suggest that skeletal muscle fibers in (*dysferlin* ^sjl/+^: *fukutin* ^Hp/−^) mice have latent membrane fragility, which is protected partially by dysferlin functions, and membrane fragility caused by synergy of reduced α-DG glycosylation and dysferlin-deficiency underlies the deteriorated phenotype of the (*dysferlin* ^sjl/sjl^: *fukutin* ^Hp/−^) mice.

We examined whether dysferlin itself and/or its interacting proteins, caveolin-3 [31] and MG53 [22], are compensatory upregulated in *fukutin* ^Hp/−^ mice. Western blot analysis showed that levels of dysferlin, caveolin-3, and MG53 were not significantly different between *fukutin* ^Hp/−^ and *fukutin* ^Hp/+^ skeletal muscle (Fig. S1A and B). Immunofluorescence analysis also showed no obvious change in dysferlin expression pattern between *fukutin* ^Hp/−^ and *fukutin* ^Hp/+^ skeletal muscle (Fig. S1C).

### Characterization of muscular dystrophic changes in (*dysferlin^sjl/sjl^*: *Large^myd/myd^*) mice

**Figure 6:**
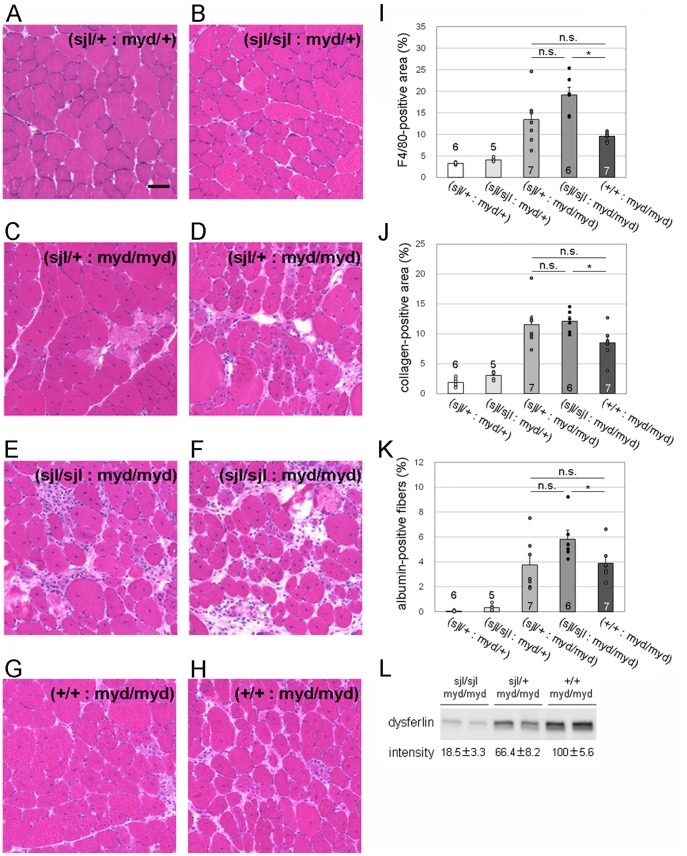
Histopathological analysis of skeletal muscle from dysferlin/Large double mutant mice. (A–H) H&E staining of TA muscle from [(*dysferlin* ^sjl/+^: *Large* ^myd/+^), A], [(*dysferlin* ^sjl/sjl^: *Large* ^myd/+^), B], [(*dysferlin* ^sjl/+^: *Large* ^myd/myd^), C and D], [(*dysferlin* ^sjl/sjl^: *Large* ^myd/myd^), E and F], and [(*dysferlin* ^+/+^: *Large* ^myd/myd^), G and H] mice at 15 weeks. Bar, 50 µm. (I) Quantitative analysis of macrophage infiltration, determined by immunofluorescence analysis using F4/80 antibody. (J) Quantitative analysis of connective tissue infiltration determined by immunofluorescence analysis using anti-collagen I antibody. (K) Quantitative analysis of the proportion of myofibers containing intracellular albumin. For quantitative analysis (I–K), data shown are mean ± s.e.m. for each group (*n* is indicated in the graph; *, *p*<0.05; n.s., not significant). (L) Western blot analysis and quantification of dysferlin expression in the total skeletal muscle lysate from (*dysferlin* ^sjl/sjl^: *Large* ^myd/myd^), (*dysferlin* ^sjl/+^: *Large* ^myd/myd^), and (*dysferlin* ^+/+^: *Large* ^myd/myd^) mice. A representative two individual samples are shown in the blot. Data shown are the average of three individual mice with standard deviations. The (*dysferlin* ^sjl/+^: *Large* ^myd/+^), (*dysferlin* ^sjl/sjl^: *Large* ^myd/+^), (*dysferlin* ^sjl/+^: *Large* ^myd/myd^), (*dysferlin* ^sjl/sjl^: *Large* ^myd/myd^), and (*dysferlin* ^+/+^: *Large* ^myd/myd^) mice are abbreviated as (sjl/+: myd/+), (sjl/sjl: myd/+), (sjl/+: myd/myd), (sjl/sjl: myd/myd), and (+/+: myd/myd), respectively.

We subsequently analyzed the histopathology of (*dysferlin* ^sjl/sjl^: *Large* ^myd/myd^) mice. *Large* ^myd/myd^ mice show severe muscular dystrophic phenotypes such as infiltration of connective and fat tissues and marked variation in fiber size [28]. Almost all α-DG is hypoglycosylated in *Large* ^myd/myd^ mice [23]. We confirmed that the pathology of (*dysferlin* ^sjl/+^: *Large* ^myd/myd^) mice was more severe than that in (*dysferlin* ^sjl/sjl^: *Large* ^myd/+^) mice (Fig. 6). To examine whether the dysferlin functions have protective roles in *Large* ^myd/myd^ skeletal muscle, we compared the pathology in (*dysferlin* ^sjl/+^: *Large* ^myd/myd^) and (*dysferlin* ^sjl/sjl^: *Large* ^myd/myd^) mice. The (*dysferlin* ^sjl/+^: *Large* ^myd/myd^) mice showed necrotic and centrally nucleated fibers, indicating frequent cycles of muscle degeneration and regeneration (Fig. 6C). In addition, some animals showed signs of advanced muscular dystrophic changes such as variations in fiber size and connective tissue infiltration (Fig. 6D). The (*dysferlin* ^sjl/sjl^: *Large* ^myd/myd^) mice exhibited severe pathology, including marked variation in fiber size and large areas with infiltration (Fig. 6E and F). We evaluated these pathologies quantitatively by measuring the areas of macrophage or connective tissue infiltration and the population of albumin-positive muscle fibers (Fig. 6I, J, and K). Both the macrophage-infiltrated area and the population of albumin-positive muscle fibers tended to be larger in (*dysferlin* ^sjl/sjl^: *Large* ^myd/myd^) than in (*dysferlin* ^sjl/+^: *Large* ^myd/myd^); however, we did not observe statistically significant differences between the two groups. Furthermore, quantification of collagen I immunofluorescence showed no significant difference in connective tissue infiltration between (*dysferlin* ^sjl/sjl^: *Large* ^myd/myd^) and (*dysferlin* ^sjl/+^: *Large* ^myd/myd^) skeletal muscles. These results suggest that dysferlin function produces limited protective effects against the progression of severe muscular dystrophy in *Large* ^myd/myd^ mice. Interestingly, however, when compared with the (*dysferlin* ^+/+^: *Large* ^myd/myd^) mice, the (*dysferlin* ^sjl/sjl^: *Large* ^myd/myd^) mice showed significant increases in F4/80, collagen I and intracellular albumin staining (Fig. 6I, J, and K). The amount of dysferlin protein in total lysates from (*dysferlin* ^sjl/sjl^: *Large* ^myd/myd^) and (*dysferlin* ^sjl/+^: *Large* ^myd/myd^) skeletal muscles was estimated to be ∼20% and ∼60% of that from (*dysferlin* ^+/+^: *Large* ^myd/myd^) muscle, respectively (Fig. 6L). These results suggest that the dramatic reduction in the amount/activity of dysferlin protein may be associated with a worse phenotype in the (*dysferlin* ^sjl/sjl^: *Large* ^myd/myd^) mice. Overall, our results suggest that the protective effects of dysferlin on dystroglycanopathy phenotype appear to be diminished when the dystrophic pathology is severe and progressive and also may depend on the amount of dysferlin proteins.

## Discussion

Here we have characterized the contribution of dysferlin-deficiency to the pathology of dystroglycanopathy using double mutant mice for dysferlin and α-DG glycosylation. To date, several dystroglycanopathy model mice have been established. *Large^myd^* mice [28] and knock-in mice carrying the FKRP P448L mutation [32] show no detectable amounts of functionally glycosylated α-DG, no laminin binding activity, and progressive muscular dystrophy. On the other hand, other dystroglycanopathy mouse models do not show a muscular dystrophy phenotype [23]. We previously reported that a small amount of intact α-DG in *fukutin* ^Hp/−^ mice is sufficient to maintain muscle cell integrity, thus preventing muscular dystrophy [23]. These results and others suggest that the presence of functionally glycosylated α-DG can decrease disease severity [33], [34]. In the present study, however, we showed that although (*dysferlin* ^sjl/+^: *fukutin* ^Hp/−^) mice did not exhibit a muscular dystrophy phenotype, (*dysferlin* ^sjl/sjl^: *fukutin* ^Hp/−^) mice developed a more exacerbated phenotype than did the *dysferlin* single-mutant (*dysferlin* ^sjl/sjl^: *fukutin* ^Hp/+^) mice. It has been widely accepted that α-DG glycosylation plays an important role in preventing disease-causing membrane fragility by maintaining a tight association between the basement membrane and the muscle cell membrane, and its defects produce muscle membrane that is susceptible to damage [24], [29]. The synergically exacerbated phenotype of the (*dysferlin* ^sjl/sjl^: *fukutin* ^Hp/−^) mice suggests latent membrane fragility in *fukutin*-deficient *fukutin* ^Hp/−^ skeletal muscle. Indeed, the increased number of intracellular albumin-positive fibers in the (*dysferlin* ^sjl/sjl^: *fukutin* ^Hp/−^) mice also supports this hypothesis. It is assumed in the *fukutin* ^Hp/−^ myofiber that interaction between the basement membrane and the cell membrane may be weakened, and therefore disease-causative membrane damage could occur during muscle contractions. However, such presumable membrane fragility may be protected in part by the dysferlin functions.

It is known that dysferlin plays a role in membrane repair pathway and several proteins are known to interact with dysferlin, suggesting that dysferlin forms a protein complex during the membrane repair process. MG53 has been shown to interact with dysferlin and participate in membrane repair, and genetic disruption of MG53 in mice results in muscular dystrophy [22]. Caveolin-3 is known to interact with dysferlin and MG53 [31], [35]. In the present study, however, we did not observe compensatory upregulation of these proteins in *fukutin* ^Hp/−^ mice, suggesting that dysferlin functions other than membrane repair may play protective roles in the *fukutin* ^Hp/−^ mice. Recently, accumulating evidence has suggested new dysferlin roles other than membrane repair, such as T-tubule formation, maintenance, and stabilizing stress-induced Ca^2+^ signaling [36], [37]. In addition, it has been reported that dysferlin deficiency leads to increased expression of complement factors and that complement-mediated muscle injury is associated with the pathogenesis of dysferlin-deficient muscular dystrophy [38]. Therefore, it is possible that such impairments independently or synergically contribute to the pathology of the double mutant mice.

Our results showed, rather unexpectedly, that the double-mutant (*dysferlin* ^sjl/sjl^: *Large* ^myd/myd^) mice did not exhibit significant deterioration of muscle pathology compared with the single-mutant (*dysferlin* ^sjl/+^: *Large* ^myd/myd^) mice. These data suggest that the protective effects of dysferlin in *Large* ^myd/myd^ mice were slightly or much reduced compared with those in *fukutin* ^Hp/−^ mice. Since *Large* ^myd/myd^ mice showed severe and rapid progressive pathology while *fukutin* ^Hp/−^ mice were asymptomatic, our data suggest that the protective effect of dysferlin may be less when disease pathology is advanced and/or severe. It has been reported that a double mutant of dysferlin and dystrophin produced a more exacerbated phenotype than did either single mutant [39]. In our colony, *Large* ^myd/myd^ mice show much more severe and rapid progressive pathology than do dystrophin-deficient mdx mice, supporting our hypothesis of a limited protective effect of dysferlin in dystrophic pathology. Interestingly, the (*dysferlin* ^sjl/sjl^: *Large* ^myd/myd^) mice, however, showed a significantly worse phenotype that did the (*dysferlin^+^* ^/+^: *Large* ^myd/myd^) mice. In addition, there is a tendency toward a worse phenotype in the order of dysferlin amount, *i.e.* (*dysferlin^+^* ^/+^: *Large* ^myd/myd^), (*dysferlin* ^sjl/+^: *Large* ^myd/myd^), and (*dysferlin* ^sjl/sjl^: *Large* ^myd/myd^). These data support the possibility that the protective effect of dysferlin is present even in the severe dystrophic *Large* ^myd/myd^ mice. We conclude that dysferlin has the potential to protect muscular dystrophy progression; however, its effect may depend on disease severity and the amount/activity of dysferlin proteins.

Recently, we showed that the retrotransposal insertion in the 3′-UTR region of *fukutin* causes abnormal mRNA splicing, which is induced by a strong splice acceptor site in SVA and a rare alternative donor site in the last exon, to produce an aberrantly spliced fukutin protein [7]. The introduction of antisense oligonucleotides that target the splice acceptor, the predicted exonic splicing enhancer, and the intronic splicing enhancer prevented the pathogenic exon trapping by SVA in the cells of FCMD patients as well as model mice (*fukutin* ^Hp/Hp^ and *fukutin* ^Hp/−^) [7]. This therapeutic strategy can potentially be applied to almost all FCMD patients in Japan, and can therefore be the first radical clinical treatment for dystroglycanopathies. However, there was no animal model to test the effectiveness of the antisense oligonucleotide therapy. Since *fukutin* ^Hp/−^ mice do not exhibit any signs of muscular dystrophy [23], they are not a great model for examining therapeutic effects of this strategy. Skeletal muscle-selective fukutin cKO mice, MCK-fukutin-cKO and Myf5-fukutin-cKO, showed dystrophic pathology [24], but they do not possess the retrotransposal insertion, and thus they are not applicable for testing the antisense oligonucleotide therapy. Our present study demonstrates more severe dystrophic phenotype of (*dysferlin* ^sjl/sjl^: *fukutin* ^Hp/−^) mice compared with (*dysferlin* ^sjl/sjl^: *fukutin* ^Hp/+^) mice. Since the (*dysferlin* ^sjl/sjl^: *fukutin* ^Hp/−^) mice possess the retrotransposal insertion and show dystrophic phenotype, they will be used as the first model for evaluation of the antisense oligonucleotide therapy for FCMD. There is a possibility that the absence of dysferlin could add hurdles on how to interpret the results of the antisense oligonucleotide treatments; however, our quantitative assessments established in this study could overcome this issue. For example, macrophage infiltration (Fig. 4B), connective tissue infiltration (Fig. 4D), and membrane fragility in quadriceps muscles (Fig. 5B) were significantly increased only in the (*dysferlin* ^sjl/sjl^: *fukutin* ^Hp/−^) mice. These parameters in the (*dysferlin* ^sjl/sjl^: *fukutin* ^Hp/+^) mice were not changed compared with those in the (*dysferlin* ^sjl/+^: *fukutin* ^Hp/+^) and the (*dysferlin* ^sjl/+^: *fukutin* ^Hp/−^) mice, and therefore can be used for quantitative evaluation for therapeutic effects of the antisense oligonucleotide treatments. We hope that generation of this novel FCMD model and establishment of the quantitative evaluation for disease severity will accelerate the future translational researches to overcome FCMD.

## Supporting Information

Figure S1 **Expression of dysferlin and dysferlin-interacting proteins in** ***fukutin*** **^Hp/−^ mice.** (A) Western blot analysis of dysferlin, caveolin-3, and MG53 in skeletal muscle extracts from fukutin-deficient *fukutin* ^Hp/−^ (Hp/−), and control *fukutin* ^Hp/+^ (Hp/+) mice. A representative two individual samples for each mouse line are shown in the blots. (B) Quantification of protein expression (panel A) was shown in graphs. Data shown are the average with standard deviations (*n* = 4 for dysferlin, *n* = 3 for caveolin-3 and MG53). (C) Immunofluorescence analysis of dysferlin in *fukutin* ^Hp/−^ (Hp/−) and *fukutin* ^Hp/+^ (Hp/+) mice. Bar, 50 µm.
(DOCX)
