# Supplementary material for: Contribution of Dysferlin Deficiency to Skeletal Muscle Pathology in Asymptomatic and Severe Dystroglycanopathy Models: Generation of a New Model for Fukuyama Congenital Muscular Dystrophy
Source: PLoS One. 2014 Sep 8;9(9):e106721. doi: 10.1371/journal.pone.0106721 (PMC4157776; doi:10.1371/journal.pone.0106721)
Supplement: Figure S1 — Expression of dysferlin and dysferlin-interacting proteins in fukutin Hp/− mice. (A) Western blot analysis of dysferlin, caveolin-3, and MG53 in skeletal muscle extracts from fukutin-deficient fukutin Hp/− (Hp/−), and control fukutin Hp/+ (Hp/+) mice. A representative two individual samples for each mouse line are shown in the blots. (B) Quantification of protein expression (panel A) was shown in graphs. Data shown are the average with standard deviations (n = 4 for dysferlin, n = 3 for caveolin-3 and MG53). (C) Immunofluorescence analysis of dysferlin in fukutin Hp/− (Hp/−) and fukutin Hp/+ (Hp/+) mice. Bar, 50 µm. (DOCX) [file pone.0106721.s001.docx]

Supporting Information

**Contribution of dysferlin deficiency to skeletal muscle pathology in asymptomatic and severe dystroglycanopathy models: generation of a new model for Fukuyama congenital muscular dystrophy**

Motoi Kanagawa, Zhongpeng Lu, Chiyomi Ito, Chie Matsuda, Katsuya Miyake, and Tatsushi Toda

**^
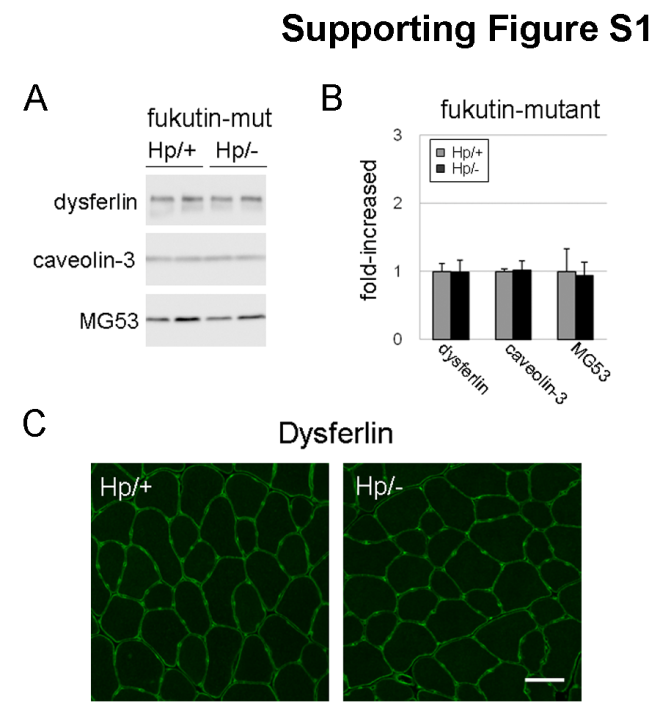
^**

**Supporting Figure S1. Expression of dysferlin and dysferlin-interacting proteins in *fukutin*^Hp/-^ mice.**

(A) Western blot analysis of dysferlin, caveolin-3, and MG53 in skeletal muscle extracts from fukutin-deficient *fukutin*^Hp/-^ (Hp/-), and control *fukutin*^Hp/+^ (Hp/+) mice. A representative two individual samples for each mouse line are shown in the blots. (B) Quantification of protein expression (panel A) was shown in graphs. Data shown are the average with standard deviations (*n* = 4 for dysferlin, *n* = 3 for caveolin-3 and MG53). (C) Immunofluorescence analysis of dysferlin in *fukutin*^Hp/-^ (Hp/-) and *fukutin*^Hp/+^ (Hp/+) mice. Bar, 50 μm.
